# The association between dietary patterns derived by three statistical methods and type 2 diabetes risk: YaHS-TAMYZ and Shahedieh cohort studies

**DOI:** 10.1038/s41598-023-27645-w

**Published:** 2023-01-09

**Authors:** Sara Beigrezaei, Sara Jambarsang, Sayyed Saeid Khayyatzadeh, Masoud Mirzaei, Amir Houshang Mehrparvar, Amin Salehi-Abargouei

**Affiliations:** 1 Nutrition and Food Security Research Center, Shahid Sadoughi University of Medical Sciences, Yazd, Iran; 2 Department of Nutrition, School of Public Health, Shahid Sadoughi University of Medical Sciences, Yazd, Iran; 3 Departments of Biostatistics and Epidemiology, School of Public Health, Center for Healthcare Data Modeling, Shahid Sadoughi University of Medical Sciences, Yazd, Iran; 4 Yazd Cardiovascular Research Center, Non-Communicable Disease Research Institute, Shahid Sadoughi University of Medical Sciences, Yazd, Iran; 5 Industrial Diseases Research Center, Shahid Sadoughi University of Medical Sciences, Yazd, Iran

**Keywords:** Diseases, Endocrinology, Medical research, Risk factors

## Abstract

Findings were inconsistent regarding the superiority of using recently introduced hybrid methods to derive DPs compared to widely used statistical methods like principal component analysis (PCA) in assessing dietary patterns and their association with type 2 diabetes mellitus (T2DM). We aimed to investigate the association between DPs extracted using principal component analysis (PCA), partial least-squares (PLS), and reduced-rank regressions (RRR) in identifying DPs associated with T2DM risk. The study was conducted in the context of two cohort studies accomplished in central Iran. Dietary intake data were collected by food frequency questionnaires (FFQs). DPs were derived by using PCA, PLS, and RRR methods considering. The association between DPs with the risk of T2DM was assessed using log-binomial logistic regression test. A total of 8667 participants aged 20–70 years were included in this study. In the multivariate-adjusted models, RRR-DP3 characterized by high intake of fruits, tomatoes, vegetable oils, and refined grains and low intake of processed meats, organ meats, margarine, and hydrogenated fats was significantly associated with a reduced T2DM risk (Q5 vs Q1: RR 0.540, 95% CI 0.33–0.87, P-trend = 0.020). No significant highest-lowest or trend association was observed between DPs derived using PCA or PLS and T2DM. The findings indicate that RRR method was more promising in identifying DPs that are related to T2DM risk compared to PCA and PLS methods.

## Introduction

Type 2 diabetes mellitus (T2DM) is an increasingly common public health concern that its prevalence remains high on the world health agenda^1^ and can cause serious damage to body systems such as kidneys, heart, eyes, as well the vascular system^2^. It is a multifactorial chronic disease emanating from interaction between genetic and lifestyle factors^3^. Lifestyle-modification studies have established that prevention of T2DM underline the major role of acquired alterations, including an unhealthy diet, sedentary behavior, overweight/obesity, tobacco use, and other environmental factors^4–8^. Moreover, T2DM is known as the most important chronic disease developed by an unhealthy modern lifestyle^9^. It has been demonstrated that the quality and quantity of diet are at the heart of T2DM pathogenesis^10^. Despite the clear effects of nutrition as a fundamental factor in the pathogenesis of T2DM, it remains unclear which dietary aspects have more impacts on its prevention and management.

Recently, the dietary pattern (DP) approach was suggested to investigate the association between diets and chronic diseases with multi-factorial etiology^11^. It is proposed that dietary patterns (DPs) can provide more information regarding the nutrition and chronic diseases link beyond the effects of foods or single nutrients^12^. Various methods have been used to derive DPs including theoretical methods (a priori), empirical methods (a posteriori), and hybrid techniques of theoretical and empirical methods^11^. A priori and a posteriori approaches are traditionally applied in DP analysis, and a frequently used posterior approach is principal component analysis (PCA)^13^. This method derives DPs by constructing uncorrelated linear combinations of original food intake variables that explain as much variation in food groups intake as possible^14^. Hence, PCA-derived patterns present actual dietary behaviors in the population; however, PCA may reveal a poor correlation with the risk of diseases because DPs related to individuals' behavior are not necessarily predictors of the disease of interest^14^.

Hybrid approaches with the combination of both a priori and a posteriori approaches, such as reduced-rank regression (RRR) and partial least squares (PLS), are proposed by researchers to derive the DP that better predict chronic diseases^13,15,16^. These methods lead to DPs that are highly correlated with a set of mediator variables between diet and disease association, called response variables. The response variables are determined based on a “priori” knowledge^13,17^. These two methods mathematically work through creating a linear combination of the predictors and response variables^17^. The RRR method strives to identify patterns through constructing linear functions of food groups, best explaining the variation in the outcomes; whereas, PLS aims to maximize the variance explained in both food groups and the responses^13^.

Few studies have assessed the association between DPs and T2DM through RRR method^18,19^. Batis et al.^18^ have suggested that using both PCA and RRR provided useful insights when studying the association of DPs with diabetes. On the other hand, no study has evaluated the DPs derived only by PLS method in association with T2DM. Moreover, one recent study found that DPs associated with adverse blood lipids are associated with incidence of T2DM^20^. Though, it is still not fully clear which approach may better predict the risk of T2DM. Therefore, we aimed to evaluate the association between DPs and T2DM risk through PCA, RRR, and PLS methods with incident T2DM, simultaneously, and also to compare the relative advantages of these methods in Iranian adults.

## Results

A total of 8667 study participants (52.5% females) had complete data to be entered to the current analysis of which 245 patients were diagnosed with T2DM after 6 years of follow-up for YaHS-TAMYZ study and 4 years for Shahedieh study. The baseline characteristics of the study population are presented in Table 1. There were significant differences between participant with and without T2DM across age categories, educational status, smoking status (P = 0.003), and total energy intake. Participants with T2DM were in higher age categories than participant without T2DM (P < 0.001). Compared to cases with T2DM, participant without T2DM were more to have high school diploma and BSc or higher academic degree (P = 0.026). In addition, participants with T2DM were more likely to be current and former smokers compared to participants without T2DM (P = 0.003). While, participants without T2DM had higher energy intake than cases with T2DM (P = 0.009). No significant difference was observed between two groups of participants for sex, marital status, BMI categories, and physical activity (P > 0.05).

**Table 1:** Baseline characteristics of study participants.

	Total participants (n = 8667)	Participants with T2DM (n = 245)	Participants without T2DM (n = 8422)	P-value
**Age category, n (%)**				< 0.001
20–29 (years)	489 (5.6)	11 (4.5)	478 (5.7)	
30–39	2267 (26.2)	37 (15.2)	2230 (26.7)	
40–49	2754 (31.8)	81 (33.2)	2673 (32)	
50–59	1934 (22.3)	65 (26.6)	1869 (22.4)	
≥ 60	1159 (13.4)	50 (20.5)	1109 (13.3)	
**Sex n (%)**				0.386
Male	4047 (46.7)	122 (49.8)	3925 (47)	
Female	4551 (52.5)	123 (50.2)	4428 (53)	
**BMI n (%)**				0.076
≤ 18.5	190 (2.2)	5 (2.1)	185 (2.3)	
18.5–24.9	2214 (25.5)	44 (18.3)	2170 (26.4)	
25–29.9	3457 (39.9)	108 (44.8)	3349 (40.8)	
30–34.9	1912 (22.1)	61 (25.3)	1851 (22.6)	
≥ 35	675 (7.8)	23 (9.5)	652 (7.9)	
**Marital status, n (%)**				0.310
Single	355 (4.1)	6 (2.4)	349 (4.1)	
Married	8006 (92.4)	228 (93.1)	7778 (92.4)	
Widow or divorced	306 (3.5)	11 (4.5)	295 (3.5)	
**Educational status, n (%)**				0.026
Uneducated	1600 (18.5)	61 (24.9)	1539 (18.3)	
Elementary or guidance school	3667 (42.3)	106 (43.3)	3561 (42.3)	
High school diploma	2027 (23.4)	46 (18.8)	1981 (23.5)	
BSc or higher academic degree	1373 (15.8)	32 (13.1)	1341 (15.9)	
**Smoking status, n (%)**				0.003
Current smoker	1048 (12.1)	27 (11)	1021 (12.1)	
Former smoker	466 (5.4)	25 (10.2)	441 (5.2)	
Never smoker	7153 (82.5)	193 (78.8)	6960 (82.6)	
Total energy intake, kcal	3175.98 ± 1207.49	2978.16 ± 1165.47	3181.74 ± 1208.27	0.009
Physical activity, MET-h/week	278.85 ± 611.37	309.85 ± 626.61	277.95 ± 610.94	0.421

*T2DM* type 2 diabetes mellitus, *BMI* body mass index, *MET* metabolic equivalent.

^a^Values are mean ± standard deviation (SD) or percentage (%).

^b^P-value are resulted from independent t-test for quantitative variables and from chi-square for qualitative variables.

The 33 dietary food groups and factor loadings for each DPs derived by PCA, PLS, and RRR methods are shown in Table 2. The first DPs derived by PCA method (PCA-DP1) was characterized by high intake of processed meats, organ meats, fish, margarine, fruit juice, pizza, snacks, sweet dessert, and soft drinks and low intake of whole grains. The PCA-DP2 was associated with high intakes of dairy products, fruits, tomatoes, other vegetables, potatoes, refined grains, and vegetable oils.in addition, and PCA-DP3 was characterized by high intake of tea, mayonnaise, nuts, hydrogenated fats, sugars, and soft drinks.

**Table 2:** Factor loadings of food groups in dietary patterns identified using PCA, PLS and RRR methods.

Food groups	PCA			PLS			RRR		
	DP 1	DP 2	DP 3	DP 1	DP 2	DP 3	DP 1	DP 2	DP 3
Processed meats	0.237	–	–	− 0.238	–	–	− 0.211	–	− 0.251
Red meats	–	–	–		–	–	− 0.284	–	–
Organ meats	0.284	–	–	− 0.249	–	–	–	–	− 0.215
Fish	0.232	–	–	− 0.226	–	–	− 0.211	–	–
Poultry	–	–	–	− 0.236	–	–	− 0.290	0.265	–
Eggs	–	–	–		–	–	–	–	–
Margarine	0.218	–	–	− 0.213	–	− 0.297	− 0.204	–	− 0.281
Dairy products	–	0.251	–	–	–	–	–	–	–
Tea	–	–	0.386	–	− 0.304	–	–	–	–
Coffee	–	–	–	–	–	–	–	–	–
Fruits	–	0.329	–	–	–	0.425	–	0.249	0.286
Fruit juice	0.255	–	–	− 0.251	–	–	− 0.229	–	0.220
Tomatoes	–	0.361	–	–	–	0.373	–	–	–
Other vegetables	–	0.393	–	–	–	0.321	–	–	–
Garlic	–	–	–	–	–	–	–	–	–
Potatoes	–	0.289	–	–	− 0.342	–	–	− 0.460	–
Whole grains	− 0.231	–	–	0.286		–	0.370		–
Refined grains	–	0.245	–	–	− 0.535	–	–	− 0.281	0.427
Pizza	0.239	–	–	− 0.244	–	–	− 0.249	–	–
Snacks	0.269	–	–	− 0.263	–	–	− 0.226	–	–
Dried fruit	–	–	–	–	–	–	–	–	–
Mayonnaise	–	–	0.211	–	–	–	–	− 0.232	–
Nuts	–	–	0.243	–	–	–	–	–	–
Olive	–	–	–	–	–	–	–	–	–
Sweet dessert	0.358	–	–	− 0.338	–	–	− 0.207	–	–
Hydrogenated fats	–	–	0.221	–	–	–	–	–	− 0.264
Vegetables oils	–	0.213	–	–	− 0.293	–	–	–	0.295
Sugars	–	–	0.488	–	− 0.400	–	–	–	–
Soft drinks	0.212	–	0.329	–	–	–	− 0.214	0.263	–
Yoghurt drink	–	–	–	–	–	0.280	–	0.333	–
Salt	–	–	–	–	–	–	–	–	–
Pickles	–	–	–	–	–	–	–	–	–
Legumes	–	–	–	–	–	–	− 0.236	–	–

Factor loadings < │0.2│ were deleted for simplicity.

*DP* dietary pattern.

Using PLS method, we also derived three DPs: (1) PLS-DP1: high intake of whole grains and low intake of processed meats, organ meats, poultry, fish, margarine, fruit juice, pizza, snacks, and sweet dessert; (2) PLS-DP2: low intake of tea, potatoes, refined grains, sugars, and vegetable oils; (3) PLS-DP3: higher intake levels of fruits, tomatoes, other vegetables, and yoghurt drink, but low intake of margarine.

The first DPs from RRR method (RRR-DP1) was rich in whole grains and low in processed meats, red meats, poultry, fish, margarine, fruit juice, pizza, snacks, sweet dessert, and soft drinks. The RRR-DP2 was characterized primarily by high intake of poultry, fruits, soft drinks, and yoghurt drink and low intake of potatoes, refined grains, and mayonnaise; and the third DPs (RRR-DP3) was defined by high intake of fruits, fruit juice, refined grains, and vegetable oils, but low intake of processed meats, organ meats, margarine, and hydrogenated fats.

The percentage of variation explained by food groups was higher in DPs derived by PCA method (23.142%) in comparison to 19.252% of PLS-derived DPs and 13.89% for RRR-derived DPs (Table 3).

**Table 3:** Explained variation in food groups and responses using PCA, PLS, and RRR.

	Explained variation in food groups			Explained variation in responses		
	PCA	PLS	RRR	PCA	PLS	RRR
DP 1	11.195	10.395	6.086	0.210	0.344	0.481
DP 2	6.783	4.882	3.563	0.049	0.259	0.292
DP 3	5.163	3.974	4.240	0.064	0.227	0.219
Total	23.142	19.252	13.89	0.324	0.831	0.993

*Food items from the food frequency questionnaire were defined as 33 separate food groups (Table 4).

**Selected response variables were waist circumference, fasting blood glucose, triglycerides, low-density lipoprotein-cholesterol, high-density lipoprotein-cholesterol, and total serum cholesterol.

The three DPs of PCA explained 0.324% of the response variables variation. DPs from PLS method explained 0.831% of the total variation in six response variables and the RRR-derived DPs explained 0.993%. As expected, both RRR and PLS methods explained a greater amount of variation in the response variables (Table 3).

**Figure 1:**
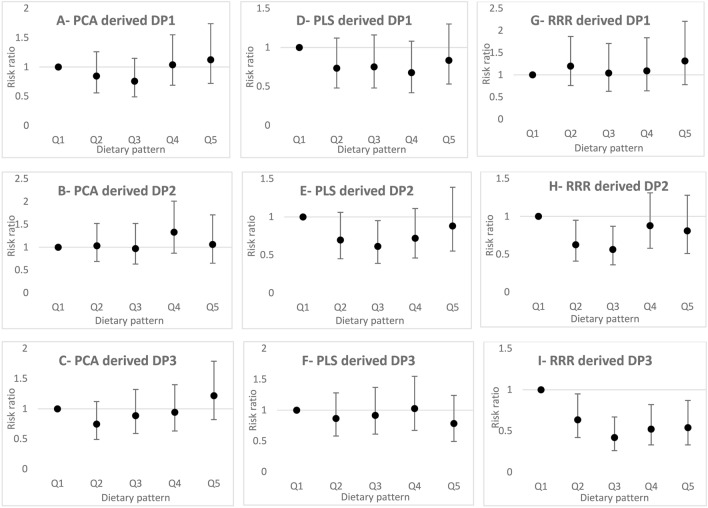
Risk ratios and 95% confidence intervals for the association between dietary patterns (DPs) derived using principal component analysis (PCA, (**A–C**)), partial least-squares (PLS, (**D–F**)), and reduced-rank regression (RRR, (**G–I**)) and type 2 diabetes mellitus (T2DM).

Figure 1 represents the risk of developing T2DM for each quintile of the DPs scores compared to the lowest quintile. No association was observed between three DPs from PCA method and T2DM risk in crude and all adjusted models.

The crude model of second DP derived by PLS method was inversely associated with T2DM risk (PLS-DP2 Q3 vs Q1: risk ratio (RR) 0.609, 95% confidence interval (CI) 0.39–0.94, P-trend = 0.585). In the multivariate-adjusted models, PLS-DP2 method was found to be inversely associated with T2DM risk in participants in the third quintile than in people in the first quintile (Model I Q3 vs Q1: RR = 0.609, 95% CI 0.39–0.94, P-trend = 0.997; Model II Q3 vs Q1: RR = 0.608, 95% CI 0.39–0.94, P-trend = 0.981; and Model III Q3 vs Q1: RR = 0.613, 95% CI 0.39–0.95, P-trend = 0.975).

In the crude models, two DPs derived by RRR method were inversely associated with T2DM risk (RRR-DP2 Q3 vs Q1: RR = 0.602, 95% CI 0.39–0.92, P-trend = 0.661; RRR-DP3 Q4 vs Q1: RR = 0.585, 95% CI 0.37–0.91 P-trend = 0.073). In the multivariate-adjusted models, RRR-DP3 was inversely associated with T2DM risk in all adjusted models (Model I Q5 vs Q1: RR = 0.599, 95% CI 0.37–0.94, P-trend = 0.035; Model II Q5 vs Q1: RR = 0.557, 95% CI 0.34–0.89, P-trend = 0.025; and Model III Q5 vs Q1: RR = 0.540, 95% CI 0.33–0.87, P-trend = 0.020). In addition, the inverse association between RRR-DP2 and T2DM risk was significant only for participants in the third quintile than those who in the lowest adherence to RRR-DP2 (Model I Q3 vs Q1: RR = 0.567, 95% CI 0.36–0.87, P-trend = 0.926; Model II Q3 vs Q1: RR = 0.568, 95% CI 0.36–0.87, P-trend = 0.950; and Model III Q3 vs Q1: RR = 0.564, 95% CI 0.36–0.87, P-trend = 0.786).

## Discussion

The greater adherence to a diet characterized by high intake of fruits, tomatoes, vegetable oils, and refined grains and low intake of processed meats, organ meats, margarine, and hydrogenated fats derived by RRR method was significantly associated with reduced T2DM risk. The present study also showed that the RRR method can provide a better identifying DPs that are related to T2DM risk due to the considering intermediate factors related to diseases for generating DPs.

Several studies have assessed the association between DPs derived only by RRR method and T2DM. Duan et al.^20^ reported that blood lipids-related DPs using the RRR method, for both men and women were characterized by high consumption of sugary beverages, juice, and added sugar; and low consumption of cereals, fruits, vegetables, nuts or seeds, and tea were significantly linked with an increased risk of T2DM. Liese et al.^21^ have used the RRR method on plasminogen activator inhibitor-1 (PAI-1) and fibrinogen biomarkers to derive DPs, and they identified a DP that was predictive of T2DM which was characterized by a high intake of red meat, fried potatoes, tomato vegetables, dried beans, low-fiber bread and cereal, eggs, cheese, and low intake of wine. A nested case–control study identified a RRR-derived DPs using inflammatory biomarkers that was characterized by a high intake of processed meats, soft drinks, sugar-sweetened drinks, and refined grains, but a low intake of cruciferous and yellow vegetables, wine, and coffee that was associated with an increased T2DM risk^22^. However, the differences in the results of our study and the aforementioned study could be influenced by the difference between responses variables.

In the current study, people in second and third quintile of adherence to RRR-DP2 had lower risk of T2DM; In addition, the inverse association between adherence to PLS-DP2 and T2DM risk was observed only in modest quintile. Whereas, no association between highest adherence to PLS-DP2 and RRR-DP2 and T2DM risk was observed.

Our findings revealed that although PCA explains the highest variation in food groups, none of the derived DPs by this method were significantly associated with T2DM risk. This supports the view that PCA generates the diet behavior-related patterns and PCA-derived DPs could not necessarily predict the risk of diseases.

Altogether, in this study, we found more T2DM-associated DPs by using the RRR method than both PLS and PCA. In line with our results, Hoffmann et al.^13^ compared three methods PCA, RRR, and PLS in association with T2DM and found that the RRR method could extract significant risk factors for T2DM. It should be considered that RRR method focuses on explaining variation in the disease-related response variables, while PLS is a method that mathematically considers both food groups and responses. This fact may explain the significant associations between RRR-derived DPs and T2DM rather than PLS-derived DPs. Moreover, in accordance with our results, some investigations demonstrated that RRR derived DPs had stronger and more statistically significant link with other outcomes than those derived using PCA and PLS^13,16,23^.

In line with this association, numerous previous studies support the link between consumption of fruits and vegetables and a decreased risk of T2DM. A meta-analysis of prospective studies found that T2DM risk reduced by 10% with increasing intakes of fruits up to 200–300 g/day^24^. A study by Nguyen et al.^25^ showed that greater intake of fruits and vegetables are related to a lower risk of T2DM. Furthermore, a review established that the intake of fruit juices can decrease the risk of chronic diseases including T2DM^26^; whereas, two meta-analyses did not proposed an association between fruit juice intake and T2DM risk^27,28^. The favorable effects of fruits and vegetables in the prevention of T2DM could be because of their high content of fiber, vitamins, minerals, antioxidants, and phytochemicals^29,30^. In addition, antioxidant phytochemicals contribute to the reduction of oxidative stress and inflammation^30^. For instance, it is shown that blueberries reduce blood glucose^31,32^ and C reactive protein^31^ and improve the insulin sensitivity^33^. Blueberries, grapes, and apples are rich in anthocyanins and quercetin^34–36^. Animal studies have shown that anthocyanins with anti-diabetic effects via glucose transporter 4 regulation^37^. Quercetin also has a protective role in reducing oxidative stress and beta-cell damage^38^. Moreover, the magnesium content of fruits and vegetables could improve insulin signaling^39^. It has been also demonstrated that the consumption of fruits and vegetables may reduce T2DM risk by decreasing adipose tissue and weight gain over time^29^. It is shown that tomatoes are beneficial for diabetic conditions due to reducing oxidative stress, inflammation, and tissue damages^40^. Tomatoes contain a wide range of antioxidants like lycopene, vitamins, and minerals^41^; as a dose–response association was observed between serum lycopene levels and T2DM^42,43^.

It has been consistently shown that processed meats increase the T2DM risk in prospective studies^44,45^. A meta-analysis by Tian et al.^46^ also revealed that the intake of processed meats is a risk factors for T2DM. It is conceivable that the high content of nitrates or nitrites in processed meats may increase the risk of T2DM^47^. Nitrosamine compounds in processed meats are formed during manufacturing or via interactions between nitrates and amino acids in the body^48^. It has been demonstrated that Nitrosamines have a toxic effect on β cells and can raise the T2DM risk^49,50^. Additionally, advanced glycation end products from processed meats can induce inflammatory mediators related to T2DM^51^. A growing body of evidence showed that DPs containing hydrogenated fats were positively associated with T2DM risk^52,53^. Trans fats are associated with an increased risk of T2DM through increasing TG levels, postprandial insulin and glucose, and reducing glucose uptake in skeletal and cardiac muscles.

There are several limitations in this study that should be considered. Although FFQs are widely used to measure usual dietary exposures and considered as a valid and reproducible nutrition science tool, they are prone to possible misreporting and misclassification of study participants which might lead to weak or null relationships. Moreover, short follow-up period and the limited number of incident T2DM cases were other limitations of our study. In addition, both YaHS-TAMYZ and Shahedieh cohort studies had less than 5-year follow-up, therefore, in the present study, the long-term effects of DPs on T2DM risk might not be revealed. In general, determining the most effective method for deriving dietary patterns related to a specific disease varies according to the study goals such as study population, selected response variables, and outcome of interest. Further studies are required to examine the generalizability of DPs derived by different methods in other populations using the similar response variables.

In conclusion, the higher adherence to a diet characterized by high intake of fruits, tomatoes, vegetable oils, and refined grains and low intake of processed meats, organ meats, margarine, and hydrogenated fats was significantly associated with reduced risk of T2DM. The findings indicate that RRR method was more promising in identifying DPs that are related to T2DM risk than PCA and PLS methods. Though, future investigations are required to approve the relative advantages of the RRR method in association with T2DM and other nutrition-related diseases.

## Materials and methods

### Study design and study population

The Yazd Health Study (YaHS) was established in September 2014 in Yazd greater area located in central Iran. In this study 9962 participants aged 20–70 years were entered in the enrollment phase. The dietary intake assessment of participants was separately collected in Taghzieh Mardom Yazd (TAMYZ) study using a validated semi-quantitative food frequency questionnaire (FFQ)^54^. The Shahedieh cohort study is a part of a large Persian multicentral study (Persian cohort) conducted on 180,000 participants in 18 various geographical areas of Iran^55^. The Shahedieh study was established in 2014 and 9977 adults aged 35 to 70 years entered to the study at baseline. Participants also filled a semi-quantitative food frequency questionnaire to report their dietary intake. Information on demographic characteristics, smoking status, physical activity, medical history was also collected in both studies. The study protocol for YaHS-TAMYZ^56^ and Shahedieh cohort^55^ are completely described elsewhere.

**Figure 2:**
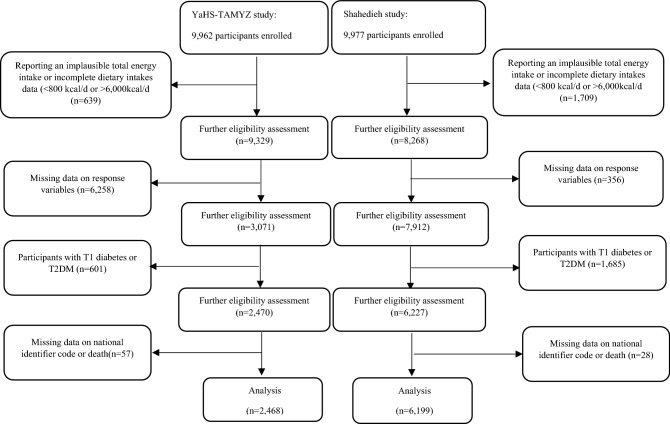
Flow chart representing the selection process of participants from YaHS-TAMYZ and Shahedieh cohort studies.

Flow chart of participant’s selection from YaHS-TAMYZ and Shahedieh cohort studies is showed in Fig. 2. Participants who reported an implausible total energy intake or incomplete dietary intakes data (< 800 kcal/day or > 6000 kcal/day, YaHS-TAMYZ study, n = 639, Shahedieh study, n = 1709), those had not provided data on response variables (YaHS-TAMYZ study, n = 6258, Shahedieh study, n = 356), those who had a previous diagnosis of type 1 diabetes or T2DM (YaHS-TAMYZ study, n = 601, Shahedieh study, n = 1685), those who had not provided data on national identifier code (YaHS-TAMYZ study, n = 34, Shahedieh study, n = 0), and people who died (YaHS-TAMYZ study, n = 23, Shahedieh study, n = 28) were excluded, which left 8667 participants (YaHS-TAMYZ: 2468, Shahedieh: 6199) for current analyses.

All participants gave an informed consent before entering the studies. Both studies were approved by the research Council of Shahid Sadoughi University of Medical Sciences. The current study was also ethically approved by Shahid Sadoughi University’s ethics committee (approval code: IR.SSU.SPH.REC.1399.197). All methods of the present study were carried out according to the relevant guidelines and regulations.

### Dietary assessment and food groups

Dietary intakes in the YaHS-TAMYZ study were assessed by a 178-item validated, multiple-choice semi-quantitative FFQ^54^. For each food item, participants were asked by trained interviewers to report the frequency of food item intake during the past year by answering 10-multiple-choice frequency responses ranging from “never or less than once a month” to “10 or more times per day”. In addition, FFQ had five choices for portion size for estimation of the amount of each consumed food item^57^. Dietary intake information was collected by a semi-quantitative open-ended FFQ based on 134-items in the Shahedieh study. Participants of the Shahedieh study were asked to report how often on average over the previous year they consumed a typical portion size of each food item with multiple possible responses on a “daily”, “weekly”, or “monthly” basis. The frequency and portion size reported for food items were converted to grams per day using household measures^58^. The United States Department of Agriculture food composition database was used to estimate daily intake of energy and nutrient for each participant^59^. Food items were merged into 33 food groups based on food items similarity in their nutrient profiles and are presented in Table 4.

**Table 4:** Food grouping used in the dietary pattern analyses in the YaHS‑TAMYZ and Shahedieh cohort studies.

Food groups	Food items
Processed meats	Sausages
Red meats	Beef, hamburger, lamb
Organ meats	Beef liver and lamb organ (tongue, tripe, head and trotters, brain, foot, abomasum)
Fish	Canned tuna fish, other fish
Poultry	Chicken with skin, chicken without skin, chicken with or without skin (liver, heart, gizzard)
Eggs	Eggs
Margarine	Margarine
Dairy products	Milk, yogurt, cheese, curd, ice‑cream, flavored milk, chocolate milk, coffee milk, honey milk, cream, butter
Tea	Tea
Coffee	Coffee
Fruits	Pears, apricots, cherries, apples, raisins or grapes, bananas, cantaloupe, watermelon, oranges, grapefruit, kiwi, grapefruits, strawberries, peaches, nectarine, tangerine, mulberry, plums, persimmons, pomegranates, lemons, pineapples, fresh figs and dates
Fruit juice	Apple juice, orange juice, grapefruit juice, other fruit juices
Tomatoes	Tomatoes, tomato pasta
Other vegetables	Cucumber, cabbage, cauliflower, brussels sprouts, kale, carrots (row or boiled), squash, spinach, lettuce, mixed vegetables (row or cooked), eggplant, celery, kohlrabi, green peas, green beans, turnip, corn, mushrooms, onions, beet, beet root, artichokes, bell pepper, pepper
Garlic	Garlic
Potatoes	Potatoes, french fries
Whole grains	Iranian dark bread (sangak, taftoon, barbari), local bread (korno, tanoori), wheat germ, oatmeal, barley, bulgur, whole grain biscuit (saghe talaee)
Refined grains	White breads (Lavash, Baguettes), noodles, pasta, rice, biscuits and wafers
Pizza	Pizza
Snacks	Potato chips, puffs, crackers, popcorn
Dried fruit	Dried figs, dried dates, dried mulberries and other dried fruits
Mayonnaise	Mayonnaise
Nuts	Peanuts, almonds, pistachios, hazelnuts, walnuts, sunflower, pumpkin and watermelon seeds
Olive	Olives and olive oils
Sweet dessert	Chocolates, cookies, cakes, confections, traditional sweets (kamache sen, poshtzik, pirashki, qottab, baqlava, loz, haji badam, nan berenj, sohan, yazdi cake), ardeh (liquid sesame), homemade halva, halva shekari (a sweet breakfast food in Iran), creme caramel, homemade cake
Hydrogenated fats	Hydrogenated fats, animal fats
Vegetables oils	Vegetables oils (except for olive oil)
Sugars	Jam, honey, sugars, candies, syrup, Nabat (an Iranian confectionery made of sugar and served by tea), noql (an Iranian confectionery), pashmak
Soft drinks	Soft drinks, non‑alcoholic beer, all types of artificial fruit juices
Yoghurt drink	Yoghurt drink
Salt	Salt
Pickles	Pickles
Legumes	Beans, peas, lima beans, broad beans, lentils, soy, split peas, mung beans

### Assessment of other covariates

The height and body weight of the study participants were measured in both YaHS-TAMYS and Shahedieh studies. In the Shahedieh study, body weight (kg) and height (cm) were measured using the National Institute of Health protocols by trained staffs. Body weight was measured while the participants were with minimum clothing and without shoes by using a digital scale (SECA, model 755, Germany). Height was measured by using a measure tape attached to a flat wall with the accuracy of 0.5 cm. In the YaHS-TAMYS study, body weight was measured by using an Omron BF511 portable digital scale (Omron Inc. Nagoya, Japan) with the accuracy of 0.1 kg, while standing on the middle of the scale, without assistance and with minimum clothing and height was measured in a standing position using a tape measure on a straight wall to the nearest centimeter. Body mass index (BMI) was calculated as weight (kg)/height squared (m^2^). Waist circumference (WC) was recorded to the nearest 0.5 cm by using non-stretch tape placed midway between iliac crest and lowest rib while participants were in the standing position. In addition, hip circumference was measured over the largest part of the buttocks, with an accuracy of 0.5 cm.

Data on age, gender, physical activity, education level, smoking status, marital status, and the history of chronic diseases was collected through a similar questionnaire in both cohort studies.

In the Shahedieh cohort study, participants were asked about their usual physical activity levels in the last year and in case they had seasonal jobs^60^. In the YaHS-TAMYZ cohort study, the short version of the International Physical Activity Questionnaire (IPAQ) was used to measure physical activity level of participants^61^. Physical activity was expressed as metabolic equivalent hours per week (MET-h/week) for all participants.

Age was classified into five categories (20–30, 30–40, 40–50, 50–60, and ≥ 60 years). Educational level was categorized into four levels (Uneducated, Elementary or guidance school, High school diploma, BSc or higher academic degree). Smoker participants were defined as current smokers, former smokers, and never smokers. Marital status was categorized into three categories (Single, Married, and Widowed or divorced).

### Laboratory measurements

Fasting blood glucose (FBG) (mg/dl), triglycerides (TG), low-density lipoprotein-cholesterol (LDL-c), high-density lipoprotein-cholesterol (HDL-c), and total serum cholesterol were measured in the YaHS-TAMYZ cohort study according to the standard laboratory protocol using Pars Azmoon kits and calibrated Ciba Corning (Ciba Corp, Basle, Switzerland) auto-analyzers. In Shahedieh cohort study, blood samples (25 mL) were collected from the participants after an overnight fasting (8–12 h). The blood samples were aliquoted into serum, buffy coat, and whole blood samples. FBG, TG, LDL-c, HDL-c, and total serum cholesterol were determined from the serum samples by an auto-analyzer (Analyzer BT1500) using Pars Azmoon standard kits.

### Statistical analysis

#### Dietary patterns analysis

Three complementary data reduction techniques, including PCA, RRR, and PLS, were used to identify DPs out of 33 food groups. In PCA method, the DPs explain as much variation as possible of the food groups. RRR method identifies linear functions of predictors (food groups) that explain as much intermediate responses variation as possible with using a covariance matrix of predictors and responses in calculating the DPs scores. The PLS method combines PCA and RRR methods and calculates DPs scores considering both the predictor and response matrices; therefore, the explained variance of both food groups and intermediate responses is expected to be between the PCA and RRR methods.

The number of DPs initially produced by PCA is constrained by the number of food groups used^62^; However, we retained just three DPs from PCA for subsequent analysis was according to the scree plot, an eigenvalue (> 1), and the interpretability of the principal DPs^63^. Varimax rotation was applied to achieve orthogonal DPs and increase the interpretability of principle DPs. Sample adequacy was checked by using the Kaisere Mayere Olkin (KMO) test.

According to previous literature, WC, FBG, TG, LDL-c, HDL-c and total serum cholesterol, were used as the intermediate response variables for PLS and RRR. Response variables were collected at the baseline of both YaHS-TAMYS and Shahedieh studies.

The SAS procedure PLS were used to conduct PLS and RRR analysis, respectively. The number of DPs derived by PLS and RRR is restricted by the number of intermediate response variables used; Therefore, six DPs were specified in each method. For both methods, we calculated the continuous DPs scores (the linear functions of food groups) in the subsequent analyses and interpretations. The first three DPs obtained by PLS and RRR was retained for further analyses because these DPs explained the largest amount of variation among the response variables.

#### Follow-up of study participants and case confirmation

In the YaHS-TAMYZ cohort study, information on death events and T2DM incidence was collected by using data from population-based registries and linked outcome information from the aggregated hospital information system (Samanah Electronici PArvandeh Salamat-SEPAS) which covers 100% of public hospitals and the majority of private hospitals in Yazd province. The data was obtained from SEPAS using the National Identifier number of each participant to link data.

During follow-up time in Shahedieh cohort study, participants received annual phone calls and follow-up questionnaires were completed in terms of the occurrence of death or the incidence of T2DM diagnosis. In case a participant had expired or had been diagnosed with T2DM, investigators followed the phone call with a house or hospital visit to perform a more follow-up and to collect copies of pertinent medical documents for further evaluation and recording. If needed, medical/physical examinations were performed to formulate a T2DM diagnosis. In addition, a verbal autopsy form validated in the Iranian population was completed during the death events. Two trained internists assessed the medical documents to determine the final T2DM diagnosis or cause of death. In case of inconsistency, a third internist conducted a final assessment of the documents to reach a final decision. The same follow-up procedures were followed in the case of self-reported T2DM incidence or death.

#### Descriptive analyses and modelling

Quantitative and qualitative variables were compared between participants who were diagnosed with and without T2DM using independent sample t-test and chi-square tests, respectively. Binomial logistic regression was used to evaluate the association between DPs derived by PCA, PLS, and RRR analyses and risk of T2DM incidence. All analyses were done in crude and three multivariable-adjusted models. The first model was adjusted for age, sex, and energy intake (Model I); Model II was additionally adjusted for education, marital status, smoking status, and physical activity; and in Model III, BMI was additionally controlled.

All statistical analyses were conducted with SAS Version 8.02 (SAS Institute, Cary, NC, USA) and R-4.2.2 (https://cran.r-project.org/bin/windows/base/). P values less than 0.05 were considered as statistically significant.

#### Comparison of dietary pattern methods

In this study, PCA, PLS and RRR methods were compared according to the relative factor loading within each DPs and its association with risk of T2DM. Additionally, these methods were evaluated based on the magnitude of variation of each method which explained the food groups and response variables.

### Ethics approval and consent to participate

YaHS-TAMYZ and Shahedieh cohort studies were approved by the research Council and the ethics committee of Shahid Sadoughi University of Medical Sciences. The present study was approved by the ethics committee of Shahid Sadoughi University of Medical Sciences (Approval Code: IR.SSU.SPH.REC.1399.197). All participants gave an informed consent before entering both studies.

## Data Availability

The data of the current study is available from the corresponding author on reasonable request.
